# Ultrasonographic features of eccrine spiradenoma: A case report

**DOI:** 10.1097/MD.0000000000025469

**Published:** 2021-04-09

**Authors:** Yuanyuan Xing, Xiaojing Wu, Caina Xu, Li Sun, Huiying Li, Yafei Zhang, Hongyuan Xue, Yuquan Ye

**Affiliations:** Department of Ultrasound, Hebei General Hospital, Shijiazhuang, Hebei, China.

**Keywords:** case report, differential diagnosis, eccrine spiradenoma, ultrasonography

## Abstract

**Rationale:**

Eccrine spiradenoma (ES) is a rare benign skin adnexal tumor originating from eccrine sweat glands. The features of ES on ultrasonography (US) have received little attention. Therefore, we report the sonographic findings in a case of an ES that originated from the abdominal wall and discuss the previously reported cases.

**Patient concerns:**

A 53-year-old woman was admitted to our hospital with a complaint of a painful nodule on the right side of her abdominal wall of 1-year duration.

**Diagnoses:**

The mass on the right side of abdominal wall was diagnosed as ES by histopathological examination.

**Interventions:**

The patient subsequently underwent total excision of the mass.

**Outcomes:**

The patient recovered well and had no complications during the 1-year follow-up.

**Lessons:**

As eccrine spiradenoma (ES) is rare and most of the tumors are excised without prior imaging studies. Little is known regarding the features of ES on ultrasonography (US). Familiarizing with the clinical and US features of this rare tumor may increase awareness of the disease among sonographers and clinicians.

## 1 Introduction

Eccrine spiradenoma (ES) is a rare benign dermal tumor originating from eccrine sweat glands and developing as a result of differentiation of the ductal and secretory components of eccrine sweat glands. Because of its rare incidence and usually excised without prior imaging studies, few cases describing the ultrasonography (US) imaging findings of ES have been published. Therefore, we report the sonographic findings in a case of an ES that originated from the abdominal wall and discuss the previously reports.

## 2 Case report

### 2.1 Patient information

A 53-year-old woman inadvertently found a painful nodule on the right side of her abdominal wall 1 year before. The size of the nodule was like a soybean, and the nodule has recently increased in size. The patient had intermittent pain that had recently worsened and hence was admitted to our hospital. Physical examination revealed a mass on the right side of the abdominal wall, which was approximately 1 cm in diameter. It was blue to purple in color, with a smooth contour, protruding from the surface of the skin, accompanied by obvious tenderness and no swelling or ulceration of the skin. She did not have any history of longstanding illness and malignancy. There was no history of any abdominal surgeries or trauma. Laboratory tests revealed normal findings.

### 2.2 Imaging manifestations

**Figure 1:**
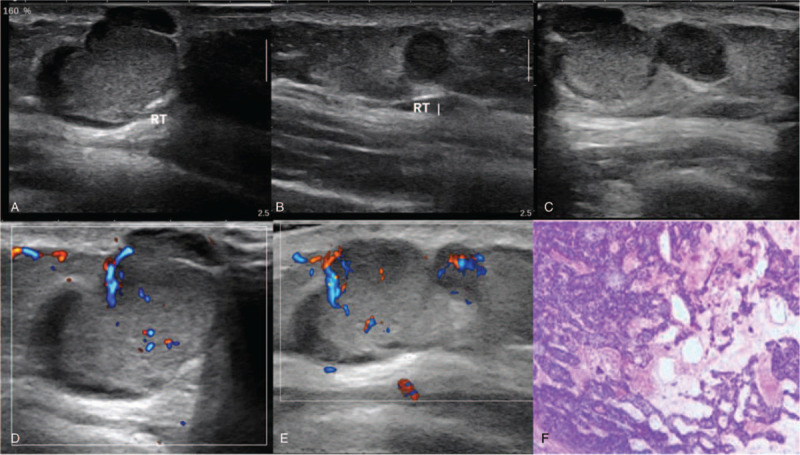
(A, B, C), Several mass lesions in the dermis and superficial subcutaneous fat layer. They showed no extension into muscle layer, with well-defined margin, heterogeneous echo texture, and posterior acoustic enhancement. The lesions were in close proximity with similar sonographic structure. (D, E), Color Doppler sonography shows blood flow signal in the central and peripheral region. (F), Histopathological section of the surgical biopsy specimen.

Longitudinal and transverse US was performed using a SuperSonic US device equipped with a SL15–4 linear probe. The sonographic images revealed several mass lesions in the dermis and superficial subcutaneous fat layer. They showed no extension into muscle layer. The larger 1 measured 1.8cm × 1.6cm × 0.8 cm, with well-defined margin, heterogeneous echo texture, and posterior acoustic enhancement. The lesions were in close proximity and showed similar sonographic structure (Fig. 1A,B,C). On color Doppler sonography, blood flow was presented in the central and peripheral region (Fig. 1D,E). US diagnosis was multiple mass lesions.

### 2.3 Histopathological results

The patient subsequently underwent total excision of the tumor. The tumor was a well-defined lobulating mass in the dermis and the subcutaneous fat layer, measuring 3.0cm × 2.5cm × 1.0 cm, which was soft, containing cystic and solid regions. Immunohistochemical staining showed: CK7 (+), CK8/18 (+), EMA (partial +), S100 (+), CEA (-), Ki-67 (approximately 15%+), p53 (++), LCA(lymphocyte+), ER(scatter+). Special staining showed: PAS (+) (Fig. 1F). The final diagnosis was ES. The patient recovered well and had no complications during the 1-year follow-up.

## 3 Discussion

ES was first reported by King in 1930. In 1956, the clinical and pathological manifestations were described in detail by Kersting and Helwing.^[1]^ ES is a rare and benign tumor of the sweat glands, located in the dermis and subcutaneous tissue. This tumor may occur at any age, but it is most frequently seen in 11 to 40 years old people and without a predilection for either gender.^[2,3]^ Congenital cases are occasionally reported. There is a special clinical manifestation in ES. It often presents as a single nodule ranging in size from 0.3 to 5 cm in diameter. Patients most often present with complaint of a painful nodule. The mass is mostly blue or skin color, may be seen as pink, red, purple, brown nodule on skin as well.^[4]^ Congenital cases are occasionally reported. ES is frequently solitary while multiple cases are often hereditary, autosomal dominant genetic diseases and have family history.^[5]^

Sonographic characteristics of ES have been reported in only a few reports. In 2008, Jin first reported the US findings of an ES in the arm.^[6]^ 2015, Balaban M first reported the sonographic and sonoelastographic findings of a benign ES.^[3]^ Then, Hwang presented a case analysis of its ultrasound performance in 2018 with the clinical data of 8 patients who underwent preoperative US at 4 different medical institutions and analyzed the US features.^[7]^ A comparative analysis of this case with the cases described in the literature was performed. In this study the ES was seen on the right side of abdominal wall compared with the literature where the location was thought to occur more commonly in the head and neck, and upper trunk by Balaban M and more commonly in the upper extremities by Hwang. This study was concordant with the first one. The ES located in dermis and the subcutaneous fat layer, without extension into the muscle layer was similar to those previously published reports. Although the sizes of ES in this case varied, all of these tumors measured only 1 or 2 cm while the literature showed that ES can range in size from 1 to 10 cm. Furthermore, compared with the imaging findings of previously reported cases, this case showed the same lobular shape and well-defined margin. However, it is worth noting that this lobular appearance was misdiagnosed as tumors by US at first which deserves more attention for diagnosis of this lesion. Moreover, this case showed hypoechoic, heterogeneous echotexture, posterior acoustic enhancement and blood flow in the central and peripheral region. These findings were also concordant with the previous reports. Ultrasonography plays a very important role in the location and differential diagnosis of ES, but the definitive diagnosis is usually based on histopathological diagnosis since the clinical and sonographic findings of the tumor are not typical. In addition, pathological diagnosis showed that the pain accompanying this tumor may be attributed to the vast nervous and capillaries plexus that surrounds the connective tissue capsule.^[8]^ Surgical resection is the curative treatment option in ES and has favorable prognosis.^[9]^ Although ES is a benign tumor and rarely shows malignant transformation, its early diagnosis and complete surgical removal are still important due to the risk of malignant transformation. Rapid growth, bleeding and ulceration in the short term indicate the possibility of malignant transformation and surgery should be performed as soon as possible.

However, ES also needs to be differentiated from other clinically painful nodules, such as schwannomas, hemangioma, angioleiomyoma, glomus tumors, skin endometriosis. Most of the schwannomas are oval and solid, and have a clear boundary and less blood flow signal. The rat tail sign, target sign are the characteristic on US.^[10]^ A hemangioma may be hypoechoic or hyperechoic relative to the surrounding tissue and have high vessel density that help in differentiating hemangiomas from ES. Angioleiomyoma appears as an oval mass with well-defined margins, homogeneous hypoechogenicity, and mainly located in the calves or ankles, parallel to the skin.^[11]^ A glomus tumor shows an oval appearance, inner hypoechogenicity, color ball sign. Most of the glomus tumors are seen at the ends of the limbs, especially under the fingernails. Skin endometriosis are usually ill-defined, irregular in shape with heterogeneous echo texture. The size and symptoms of this tumor are always related to the menstrual cycle.^[12]^

In this study, we reported a case of an ES that originated from the abdominal wall. ES is a rare benign tumor and usually excised without prior imaging studies. Therefore, its US characteristics has received little attention. Although the definitive diagnosis is usually based on histopathological diagnosis, US is useful in localization and differential diagnosis. We have learned that ES is usually located in the subcutaneous fat layer, with well-defined margin, lobulated appearance, heterogeneous echo texture, and posterior acoustic enhancement. The color Doppler flow is present in the central and peripheral region. To summarize, familiarizing with the clinical and US features of this rare tumor may increase awareness of the disease among sonographers and clinicians.

## Author contributions

**Conceptualization:** Yuanyuan Xing, Hongyuan Xue, Yuquan Ye.

**Formal analysis:** Yuanyuan Xing.

**Investigation:** Yuanyuan Xing, Xiaojing Wu, Caina Xu.

**Resources:** Yuanyuan Xing.

**Writing – original draft:** Yuanyuan Xing.

**Writing – review & editing:** Yuanyuan Xing, Li Sun, Huiying Li, Yafei Zhang.

## References

[1] Kersting DW Helwig EB. Eccrine spiradenoma. AMA Arch Derm 1956;73:199–227. 13291892 10.1001/archderm.1956.01550030001001

[2] Nath AK Kumari R Thappa DM. Eccrine spiradenoma with chondroid syringoma in Blaschkoid distribution. Indian J Dermatol Venereol Leprol 2009;75:600–2. 19915242 10.4103/0378-6323.57723

[3] Balaban M Idilman IS Unal O. Sonographic and sonoelastographic findings of a rarely seen soft tissue tumor: eccrine spiradenoma. J Med Ultrasonics 2015;42:587–90. 10.1007/s10396-015-0636-2 26576987

[4] Kim J Yang HJ Pyo JS. Eccrine spiradenoma of the scalp. Arch Craniofac Surg 2017;18:211–3. 29090205 10.7181/acfs.2017.18.3.211 PMC5647852

[5] Portincasa A Cecchino L Trecca EMC. A rare case of Brooke-Spiegler syndrome: integrated surgical treatment of multiple giant eccrine spiradenomas of the head and neck in a young girl. Int J Surg Case Rep 2018;51:277–81. 30241087 10.1016/j.ijscr.2018.08.021 PMC6146587

[6] Jin W Kim GY Lew BL. Sonographic findings of an eccrine spiradenoma: case report and literature review. J Ultrasound Med 2008;27:813–8. 18424661 10.7863/jum.2008.27.5.813

[7] Hwang CM Kang BS Hong HJ. Ultrasonographic features of eccrine spiradenoma. J Ultrasound Med 2018;37:1267–72. 29120034 10.1002/jum.14460

[8] Park HR Im SB Kim HK. Painful eccrine spiradenoma containing nerve fibers: a case report. Dermatology 2012;224:301–6. 22777362 10.1159/000339768

[9] Ter Poorten MC Barrett K Cook J. Familial eccrine spiradenoma: a case report and review of the literature. Dermatol Surg 2003;29:411–4. 12656824 10.1046/j.1524-4725.2003.29096.x

[10] Yang F Chen XX Wu HL. Sonographic features and diagnosis of peripheral schwannomas. J Clin Ultrasound 2017;45:127–33. 28090635 10.1002/jcu.22438

[11] Zhang JZ Zhou J Zhang ZC. Subcutaneous angioleiomyoma: clinical and sonographic features with histopathologic correlation. J Ultrasound Med 2016;35:1669–73. 27371376 10.7863/ultra.15.06056

[12] Koninckx PR Ussia A Wattiez A. Risk factors, clinical presentation, and outcomes for abdominal wall endometriosis. J Minim Invasive Gynecol 2018;25:342–3. 29225088 10.1016/j.jmig.2017.11.022

